# Taxonomic notes on the genus *Bellenden* Chandler (Coleoptera, Staphylinidae, Pselaphinae)

**DOI:** 10.3897/zookeys.638.10958

**Published:** 2016-12-07

**Authors:** Zi-Wei Yin, Ri-Xin Jiang

**Affiliations:** 1 Department of Biology, College of Life and Environmental Sciences, Shanghai Normal University, 100 Guilin Road, Shanghai, 200234, P. R. China

**Keywords:** Pselaphini, new synonym, new combination, new species, China, Sichuan

## Abstract

The monotypic genus *Buobellenden* Yin & Nomura, 2009 is placed as a junior synonym of *Bellenden* Chandler, 2001. This act results in *Bellenden jingyuanensis* (Yin & Nomura), **comb. n.** (from *Buobellenden*). A new species, *Bellenden siguniang* Yin & Jang, **sp. n.**, collected from the alpine area in Sichuan, SW China, is described, illustrated, and distinguished from all congeners. A new illustration of the aedeagus of *Buobellenden jingyuanensis* is given.

## Introduction

The genus *Bellenden* Chandler of the pselaphine tribe Pselaphini contains four species with a highly disjunct distribution: *Bellenden monteithi* Chandler (type species) occurs in Queensland, NE Australia, while *Bellenden belousovi* Kurbatov, *Bellenden botellarius* Kurbatov, and *Bellenden nubigena* Kurbatov are distributed in alpine regions in SW and NW China (Chandler 2001; Kurbatov 2006). Within Pselaphini, *Bellenden* shares with many genera the elongate maxillary palpomeres II and IV, but its palpomere IV is broadening throughout the entire length, rather than lengthily pedunculate or distinctly narrowed at the base. In Yin et al. (2009), a new genus *Buobellenden* Yin & Nomura was established from a single species collected in Ningxia, NW China, and was separated from *Bellenden* solely based on the aedeagal structures. Otherwise these two genera are morphologically similar. In this paper *Buobellenden* is placed as a junior synonym of *Bellenden*, and a new species of the genus is described from Sichuan, SW China.

## Material and methods

The type material of the new species is housed in the Insect Collection of Shanghai Normal University, Shanghai, China (**SNUC**).

The collecting data of the material are quoted verbatim. The Chinese translation of each locality is included in parentheses at the first appearance in the text. Each type specimen bears the following label: ‘HOLOTYPE (red) (or PARATYPE (yellow)), ♂ (or ♀), *Bellenden siguniang* sp. n., det. Yin & Jiang, 2016, SNUC’.

## Taxonomy

### Bellenden

Taxon classificationPlantaeColeopteraStaphylinidae

Chandler, 2001

Bellenden Chandler, 2001: 504; Kurbatov 2006: 361 (revision). Type species. Bellenden monteithi Chandler.
Buobellenden Yin & Nomura, 2009 (in: Yin et al. 2009: 65); **syn. n.** Type species. Buobellenden jingyuanensis Yin & Nomura.

#### Comments.

*Buobellenden* was described from a single male collected from the northwestern Chinese province of Ningxia (Yin et al. 2009). The authors compared the type species *Buobellenden jingyuanensis* with the Australian *Bellenden monteithi*, and separated these two genera based on the aedeagal characters. However, the earlier published revision of *Bellenden* by Kurbatov (2006) was not cited, in which three new *Bellenden* species were described from the alpine regions in central China. Kurbatov in his work specifically discussed the morphological differences between the *Bellenden* species from China and Australia, and concluded that creation of a new generic taxon for the Chinese *Bellenden* species is unjustified. After a re-examination of the type material of *Buobellenden*, we agree with Kurbatov’s opinion, and here place *Buobellenden* as a junior synonym of *Bellenden*.

### Bellenden jingyuanensis

Taxon classificationPlantaeColeopteraStaphylinidae

(Yin & Nomura) comb. n.

Fig. 1D

Buobellenden jingyuanensis Yin & Nomura, 2009 (in: Yin et al. 2009: 66).

#### Type locality.

Dongshanpo, Liupanshan National Nature Reserve, Jingyuan County, northwestern China.

#### Type material examined.

Holotype ♂, labeled ‘China: Ningxia A. R., Jingyuan Coun. (泾源县), (Liupanshan National Nature Reserve (六盘山自然保护区)), Dongshanpo (东山坡), alt. 2,200 m, 23.vi.2008, (Berlese Funnels), Yun Bu leg.’ (SNUC).

#### Comments.

**Figure 1.**
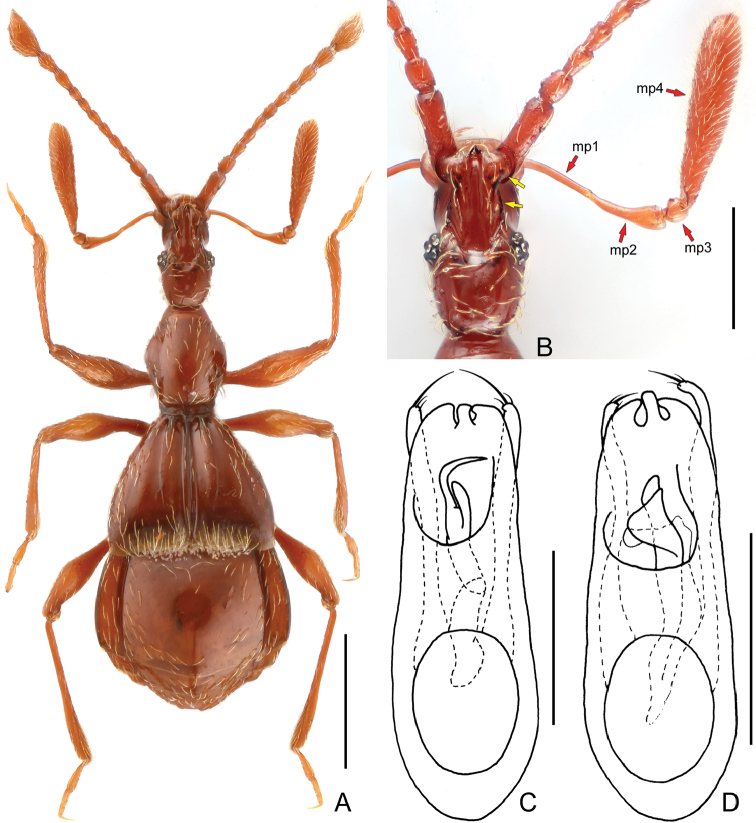
*Bellenden siguniang* (**A–C**) and *Bellenden jingyuanensis* (**D**). **A** male habitus **B** head and right maxillary palpus (yellow arrows indicate anterolateral margin of the frontal rostrum; red arrows indicate segmentation of the maxillary palpus) **C–D** aedeagus, in dorsal view. Abbreviation: mp1–4 = maxillary palpomeres I–IV. Scales bars **A** 0.5 mm; **B** 0.3 mm; **C–D** 0.2 mm.

This species can be separated from all congeners by the following combination of characters: 1) relative small body size (1.89 mm), 2) lateral margins of the frontal rostrum narrowing anteriorly, and roundly dilating laterally at the antennal bases, 3) unmodified protrochanter and metaventrite, and 4) unique structures of the aedeagal endophallus (Fig. 1D).

### Bellenden siguniang

Taxon classificationPlantaeColeopteraStaphylinidae

Yin & Jiang sp. n.

http://zoobank.org/AAB5A9CF-5316-4CEE-AF71-911C5EB4ED50

Fig. 1A–C

#### Type material

(1 ♂, 5 ♀♀)**. Holotype: CHINA**: ♂, labeled ‘China: Sichuan, Xiaojin County (小金县), Siguniang Shan (四姑娘山), Haizigou (海子沟), 30°59'57"N, 102°50'51"E, leaf litter, sifted, 3,340 m, 17.vii.2015, J(iang), P(eng), T(u), Z(hou) leg.’ (SNUC). **Paratypes: CHINA**: 5 ♀♀, same collecting data as the holotype (SNUC).

#### Diagnosis.

Lateral margins of frontal rostrum before eyes sloping anteriorly, roundly dilating laterally at level of antennal bases; maxillary palpomere I only slightly shorter than II; male lacking ventral protuberance at ventral margin of the protrochanter, metaventrite unmodified; impressed area of sternite IV longer than wide and almost reaching posterior margin of the segment.

#### Description.

Male (Fig. 1A), length 2.35 mm (combined length of head, pronotum, elytra and abdomen), body uniformly reddish-brown, pubescence recumbent and sparse. Head longer than wide, length from anterior clypeal margin to occipital constriction 0.47 mm, width across eyes 0.27 mm (Fig. 1B), frontal rostrum conspicuously narrowing anteriorly in front of anterior margin of eyes and dilating at level of antennal bases (Fig. 1B); anterior margin of frons with small triangular projection at middle, frontal sulcus without dense apical pubescence, narrow and deep anteriorly and somewhat dilating and gradually disappearing posteriorly; each eye with nine small facets; maxillary palpus (Fig. 1C) elongate, palpomere II longer than palpomere I; length of palpomere 1.05 mm, palpomere I 0.2 mm, II 0.27 mm, III 0.07 mm, IV 0.51 mm. Length of antenna 1.3 mm; antennomere I subcylindrical, more than three times as long as wide, slightly wider than antennomere II; antennomere II about 1.5 times as long as wide; antennomeres III–VIII of subequal width, slightly narrower than antennomere II, antennomeres III–VII approximately twice as long as wide, antennomere VIII slightly shorter, and antennomere IX considerably wider than preceding segments, approximately as wide as antennomere II, but much longer, approximately twice as long as wide; antennomere X somewhat slightly wider than antennomere IX, antennomere XI shorter than antennomeres IX and X together, but considerably wider than them, widest slightly before middle. Pronotum widest near middle, length along midline 0.48 mm, maximum width 0.38 mm; antebasal fovea small, puncture-like, lateral foveae larger. Elytra wider than long, length along suture 0.58 mm, maximum width 0.78 mm, covered with relatively dense long hairs along posterior margin; sutural and discal striae narrowed at base; sutural stria conspicuously deeper at base than discal stria; discal stria extending posteriorly to 3/4 elytral length. First tergite largest, widest near middle. Length of dorsally visible part of abdomen (posterior to elytra) along midline 0.82 mm, maximum width 0.92 mm; sternite IV (second visible sternite) slightly impressed medially. Aedeagus (Fig. 1C) slightly asymmetric dorso-ventrally, length 0.48 mm.

Female, similar to male in general appearance; each eye composed of nine small facets. Measurements of body parts: body length 2.24 mm, length of head 0.44–0.46 mm, width of head 0.27–0.29 mm, length of antenna 1.19–1.20 mm, length of pronotum 0.43–0.44 mm, width of pronotum 0.38 mm, length of elytra 0.59–0.61 mm, width of elytra 0.77–0.80 mm, length of abdomen 0.75–0.76 mm, width of abdomen 0.87 mm.

#### Comparative notes.

*Bellenden siguniang* is similar to *Bellenden botellarius* and *Bellenden jingyuanensis* by sharing unmodified male protrochanter and metaventrite. The new species can be separated from *Bellenden botellarius* by the lateral margins of the frontal rostrum before eyes gradually sloping anteriorly and then roundly dilating at antennal bases, and from *Bellenden jingyuanensis* by the much large body size. The lateral margins of the frontal rostrum in *Bellenden botellarius* are parallel-sided and barely dilated at antennal bases, and the body length of *Bellenden jingyuanensis* measures only 1.89 mm. *Bellenden jingyuanensis* similarly has the lateral margins of the frontal rostrum narrowing anteriorly, when combined with the structures of the aedeagal endophallus, it can be readily distinguished from *Bellenden botellarius*. The other three species, *Bellenden monteithi*, *Bellenden belousovi* and *Bellenden nubigena*, all have spinose/protuberant protrochanter in the male, thus are easily separable from *Bellenden siguniang*. Moreover, the aedeagal endophallus of *Bellenden siguniang* has a pointed and curved apex, which alone can be used to discriminate the new species from all other congeners.

#### Distribution.

Southwestern China: Sichuan.

#### Etymology.

The specific epithet is taken from the type locality of the new species, i.e., Siguniang Mountain.

## Supplementary Material

XML Treatment for Bellenden

XML Treatment for Bellenden jingyuanensis

XML Treatment for Bellenden siguniang
